# First Lunatic Asylum in the United States

**Published:** 1845-02

**Authors:** 


*First Lunatic Asylum in the United States.*—This was in the city of New York, and on the precise spot where now stands the City Hall. It was erected above one hundred years since, and consisted of a building sixty feet by twenty-four, two stories high. Into this was received the indigent poor, the sick, the orphan, the *maniac*, and the refractory. Dr. John Van Buren was the first Physician. His salary was one hundred pounds a year, he finding medicine.—*Ibid.*

